# Synthesis of Boron-Doped Zinc Oxide Nanosheets by Using *Phyllanthus Emblica* Leaf Extract: A Sustainable Environmental Applications

**DOI:** 10.3389/fchem.2022.930620

**Published:** 2022-07-12

**Authors:** Awais Khalid, Pervaiz Ahmad, Saleh Muhammad, Abdulhameed Khan, Mayeen Uddin Khandaker, Md Mottahir Alam, Mohd Asim, Israf Ud Din, Jibran Iqbal, Ibad Ur Rehman, Zohaib Razzaq, Sivakumar Pandian, Rohit Sharma, Talha Bin Emran, M. I. Sayyed, Saad Aldawood, Abdelmoneim Sulieman

**Affiliations:** ^1^ Department of Physics, Hazara University Mansehra, Mansehra, Pakistan; ^2^ Department of Physics, University of Azad Jammu and Kashmir, Muzaffarabad, Pakistan; ^3^ Department of Biotechnology, University of Azad Jammu and Kashmir, Muzaffarabad, Pakistan; ^4^ Center for Applied Physics and Radiation Technologies, School of Engineering and Technology, Sunway University, Subang Jaya, Malaysia; ^5^ Department of General Educational Development, Faculty of Science and Information Technology, Daffodil International University, Dhaka, Bangladesh; ^6^ Department of Electrical and Computer Engineering, King Abdulaziz University, Jeddah, Saudi Arabia; ^7^ Department of Chemistry, Faculty of Science, University of Jeddah, Jeddah, Saudi Arabia; ^8^ Department of Chemistry, College of Science and Humanities, Prince Sattam Bin Abdulaziz University, Al-Kharj, Saudi Arabia; ^9^ College of Natural and Health Sciences, Zayed University, Abu Dhabi, United Arab Emirates; ^10^ School of Petroleum Technology, Pandit Deendayal Energy University, Gandhinagar, India; ^11^ Department of Rasa Shastra & Bhaishajya Kalpana, Faculty of Ayurveda, Institute of Medical Sciences, Banaras Hindu University, Varanasi, India; ^12^ Department of Pharmacy, BGC Trust University Bangladesh, Chittagong, Bangladesh; ^13^ Department of Pharmacy, Faculty of Allied Health Sciences, Daffodil International University, Dhaka, Bangladesh; ^14^ Department of Physics, Faculty of Science, Isra University, Amman, Jordan; ^15^ Physics and Astronomy Department, College of Science, King Saud University, Riyadh, Saudi Arabia; ^16^ Department of Radiology and Medical Imaging, Prince Sattam Bin Abdulaziz University, Al-Kharj, Saudi Arabia

**Keywords:** bio nanoparticles, bioremediation, environmental applications, green synthesis, nanobiotechnology, anti-bacterial, boron-doping

## Abstract

The use of *Phyllanthus emblica* (gooseberry) leaf extract to synthesize Boron-doped zinc oxide nanosheets (B-doped ZnO-NSs) is deliberated in this article. Scanning electron microscopy (SEM) shows a network of synthesized nanosheets randomly aligned side by side in a B-doped ZnO (15 wt% B) sample. The thickness of B-doped ZnO-NSs is in the range of 20–80 nm. B-doped ZnO-NSs were tested against both gram-positive and gram-negative bacterial strains including *Staphylococcus aureus, Pseudomonas aeruginosa, Klebsiella pneumonia,* and *Escherichia coli*. Against gram-negative bacterium (*K. pneumonia* and *E. coli*), B-doped ZnO displays enhanced antibacterial activity with 26 and 24 mm of inhibition zone, respectively. The mass attenuation coefficient (MAC), linear attenuation coefficient (LAC), mean free path (MFP), half-value layer (HVL), and tenth value layer (TVL) of B-doped ZnO were investigated as aspects linked to radiation shielding. These observations were carried out by using a PTW^®^ electron detector and VARIAN^®^ irradiation with 6 MeV electrons. The results of these experiments can be used to learn more about the radiation shielding properties of B-doped ZnO nanostructures.

## Introduction

Scientists have been motivated by the growing environmental issues to avoid using toxic materials that could pose a severe ecological impact. As a result, researchers have recently been looking for novel solutions that are more environmentally friendly and sustainable (Adil et al., 2015). Various techniques, including physical and chemical techniques, can be used to synthesize nanomaterials. Alternative approaches, such as green chemistry, have been developed as a result of the challenges in scaling-up physical processes and the use of harmful synthetic chemicals that could be carried over by the nanostructures in chemical processes. Synthesis of nanomaterials using the green chemistry approach is started recently, while these approaches were used in agriculture, consumer items, and health for several years (Marslin et al., 2018). The green chemistry approach is based on a redox reaction in which the components of an organism or its extract convert metal ions to stable nanostructures. Plant extract-mediated synthesis of nanostructures has gained wide acceptance due to its eco-friendly nature, simplicity, easiness to scale up and low cost (Kumar and Yadav, 2009; Marslin et al., 2015).

Zinc oxide (ZnO) is such an important material that has plenty of uses and applications in almost any field of modern technology both in bulk and nano. ZnO, with a wurtzite crystal structure, is a naturally occurring wide-bandgap (3.44 eV) semiconductor material (Takahashi et al., 2007). It is an n-type semiconductor by nature having a hexagonal wurtzite phase and is known to be the most stable phase of ZnO. Because of its stability, ZnO is a good choice for electrically conductive materials (Banerjee and Guha, 1991; Borysiewicz, 2019; Wojnarowicz et al., 2020). Transparency, high conductivity, and electron mobility are all some of the excellent properties of ZnO (Ilican et al., 2011). Because of its high excitation binding energy, it can act as a transparent conductive oxide (TCO) at ambient temperature. In comparison to other wide bandgap semiconductors, ZnO has three times bigger (60 MeV) exciton binding energy (Liang and Yoffe, 1968; Look, 2001). ZnO is a versatile material and doping has a substantial impact on the optical and electrical properties, making it ideal for many applications such as piezoelectric and ferroelectric layers, UV lasers at room temperature, optoelectronic devices at short wavelength (Banerjee and Guha, 1991; Ilican et al., 2011), spintronic devices, dielectric or insulating layers, transparent conducting electrodes and radiation shielding (Hiramatsu et al., 1998; Kluth et al., 2003).

Bacterial infections are considered a severe health problem around the world. Novel bacterial mutations, pathogenic strain outbreaks, antibiotic resistance, and other factors are on the rise, necessitating the invention of more effective antibacterial agents. Antibacterial activities of ZnO have been documented earlier from time to time (Frederickson et al., 2005). ZnO is an important mineral for humans, and when given in controlled doses, it has high activity and can therefore be a good alternative for antibiotics (Zhang et al., 2007). ZnO antibacterial properties can be used in the preservation of packaged foods (Chitra and Annadurai, 2013). ZnO has been shown to prevent the intestinal tract and stomach from *E. coli* infection (Yamamoto et al., 2004). ZnO-NPs antibacterial activity depends directly on their concentration and varies inversely to their size (Raghupathi et al., 2011; Ali et al., 2021).

Boron (B) is found in both diet and the environment. B supplements are used to treat osteoarthritis, boost cognitive abilities, strengthen bones and muscles. As a result, boron (B) doping in ZnO will be very effective for a variety of biomedical applications. It has the potential to bring about a medical revolution. The as-produced B-doped ZnO thin films are found to be better antibacterial agents than pure ZnO (Kayani et al., 2020). Radiation sources are commonly used in various areas including radiation treatment centers and nuclear power plants etc. (Salinas et al., 2006; Gurler and Akar Tarim, 2012). Since radiation is used in diagnosis and treatment centers, therefore, a shield against it should be built to safeguard patients and the workers who work there. Despite all the efforts, we cannot eliminate radiation from our daily lives. As a result, basic rules such as distance, time, and shielding should be followed to mitigate the consequences of radiation (El-Khayatt et al., 2014; Agar, 2018). Precautionary measures in radiation applications are needed in a variety of disciplines (Sarachai et al., 2018). ZnO properly doped with an element having excellent properties for the desired shielding could be the best option in this regard. In such a case, B-doping could be most effective from group A-III elements of the periodic table. B-doped ZnO is a radiation shielding material famous for its clear or transparent nature. Such material is particularly desired in radiation treatment and diagnostic centers as radiation-retaining glasses indoors (for example in X-ray rooms as shielding materials), and spectacles. B-doping is mainly performed on an n-type semiconductor, which results in a larger carrier density and hence a higher tunneling current (Steinhauser et al., 2008). Boron has the smallest ionic radius (0.23 Å), as well as the greatest electronegativity (2.04, Pauling). Furthermore, B^3+^ (10.7) has a significantly greater Lewis’s acid strength compared to Al^3+^ (3.04). As a consequence, doping boron may be beneficial in fine-tuning the physical properties of ZnO nanostructures (Caglar et al., 2011). Many fabrication techniques have been used for the synthesis of B-doped ZnO in the past including radiofrequency (Rf) magnetron sputtering (Minami et al., 1985), chemical vapor deposition (CVD) (Dong et al., 2005), electrochemical deposition, atomic layer deposition (Hu and Gordon, 1992a), wet chemical synthesis, etc. (Ishizaki et al., 2002).

Many researchers including Murali et al. (Murali et al., 2021), Loganathan et al. (Loganathan et al., 2018), Chellappa et al. (Joel and Badhusha, 2016), and Shubha et al. (Shubha et al., 2019), have synthesized ZnO nanostructures using *P. emblica* plant extract and in literature, no study reported for the synthesis of B-doped ZnO-NSs synthesized using *P. emblica* plant extract. In the current study, B-doped ZnO-NSs were prepared via the unpretentious cost-effective green chemistry technique by using *P. emblica* (gooseberry) leaf extract along with zinc chloride and Boron-10. The as-prepared B-doped ZnO-NSs were used to investigate their role in antibacterial and radiation shielding applications. Free radicals are produced as a result of ionizing processes at the start of radiation exposure, and they are capable of damaging normal tissues. The pure and B doped ZnO-NSs propose a biological free radical scavenger or antioxidant action. By virtue of their antioxidant characteristics, which occur when the nanostructures penetrate the cells, the unique structure of B doped ZnO-NSs will be helpful in enhancing cell lifetime and reducing toxic exposures by reducing the formation of reactive oxygen species (ROS) and therefore inhibiting the activation of the apoptotic response and cell death.

## Materials and Methods

### Materials

Zinc dichloride (ZnCl_2_), and Boron (B^10^) of 99.9% purity was procured from Merck. The purchased chemicals were used in their original form. All the synthesis procedures were carried out with deionized water.

### Preparation of Leave Extract

Fresh leaves of *Phyllanthus emblica* (gooseberry) were collected from district Mansehra, Khyber Pakhtunkhwa, Pakistan. The collected leaves were washed gently with fresh and clean tap water thrice and then placed under sunlight for 30 min for drying. Dried leaves were cut into small pieces of the relatively same size and weighted by using a digital balance. 10 g of fine pieces of leaves were taken into a beaker containing 100 ml of de-ionized water. The beaker was then covered with aluminum foil and placed on the hotplate for 40 min. The temperature of the hotplate was set to 80°C. The mixed solution of leaves was then cooled for 20 min at room temperature where its color was found to change from green to blackish green. Whatman Grade-1 filter paper has been used for filtering the solution. The filtered solution was then centrifuged for 15 min at a rate of 4,000 rpm to settle down the dense pollutants at the bottom of the tube. The final product was collected in separate sterilized glass vials for further study of the experiment.

### Synthesis of Pure and B Doped ZnO Nanosheets

Pure ZnO and B-doped ZnO-NSs (15 wt% B) were prepared using a simple green chemistry route. ZnCl_2_ (1.7 g) was used as a starting reagent dissolved in 100 ml of de-ionized water along with 0.5 ml of HNO_3_. Then, 5 ml (ml) of the as-prepared (10 ml of leaves extract diluted with 10 ml of deionized water) leaves extract was poured drop by drop into the salt solution. The same procedure was followed to prepare B-doped ZnO-NSs using B^10^ (0.25 g) as a dopant source. The pH of the solution was noted as 4. Afterward, the solution was heated and stirred gently at 100°C for 2 h. The past-like product was then washed several times with distilled water and ethanol, and dried in a drying oven at 120°C for 1 hour. The dried powder of the synthesized B-doped ZnO-NSs was collected in an ultrafiltration vial.

### Characterization

Scanning electron microscopy (MAIA3 TESCAN), X-ray diffraction (Bruker D8 (Germany)), Energy Dispersive X-Ray Analysis (Oxford INCA X-sight 200), Fourier-transform infrared (Nicolet Avatar 370), Photoluminescence analysis (Hitachi F-4500, Japan) and Linear accelerator (VARIAN^®^) were used to study morphology, crystallographic structure, elemental analysis, structural defects, the role of biomolecules in reduction and radiation shielding behavior of the as-synthesized B-doped ZnO nanomaterial.

### Antibacterial Activity

The bactericidal potential of B-doped ZnO-NSs was determined by using the agar well diffusion method against microorganisms including *Pseudomonas aeruginosa, Klebsiella pneumonia, Escherichia coli* (Gram-negative), and *Staphylococcus aureus* (Gram-positive). All sample stock solutions have been made in dimethyl sulfoxide (DMSO) by adding the required amounts of pure ZnO and B-doped ZnO-NSs to achieve the homogeneous solution. DMSO was used to make working dilutions from stock solutions of 0.25 mg/ml, 0.5 mg/ml, and 0.75 mg/ml. To make culture media, agar nutrient was dissolved in deionized water. For sterilization, it was autoclaved for 20 min at 120°C. After cooling, the purified media was placed in 90 mm Petri dishes (autoclaved) under controlled and aseptic experimental conditions. At room temperature, the agar was allowed to firm before being stored in the refrigerator. The bacteria including *S. aureus*, K. pneumonia, *P. aeruginosa*, and *E. coli* were sub-cultured on the agar plates with the use of an autoclaved immunizing needle by moving a very minimal quantity of bacterial isolate to the agar plates to investigate the antibacterial activity. To examine for bacterial growth and contamination, the plates were incubated at 37°C for 24 h. Cotton swabs were used to inoculate the Petri plates with the bacterial inoculum. Following this, all of the plates are arranged in a laminar airflow chamber in an ordered manner for 15 min. Then using a disinfected corn borer, 4 wells with a diameter of 8 mm were created within every Petri plate, and 40 µl of each sample were poured into the corresponding well using a micropipette. To check for bacterial growth, the plates were incubated at 37°C for 24 h. After incubation, the antimicrobial effect of all samples was evaluated in millimeters by the diameter of the inhibitory zone.

### Radiation Shielding Studies

**FIGURE 1:**
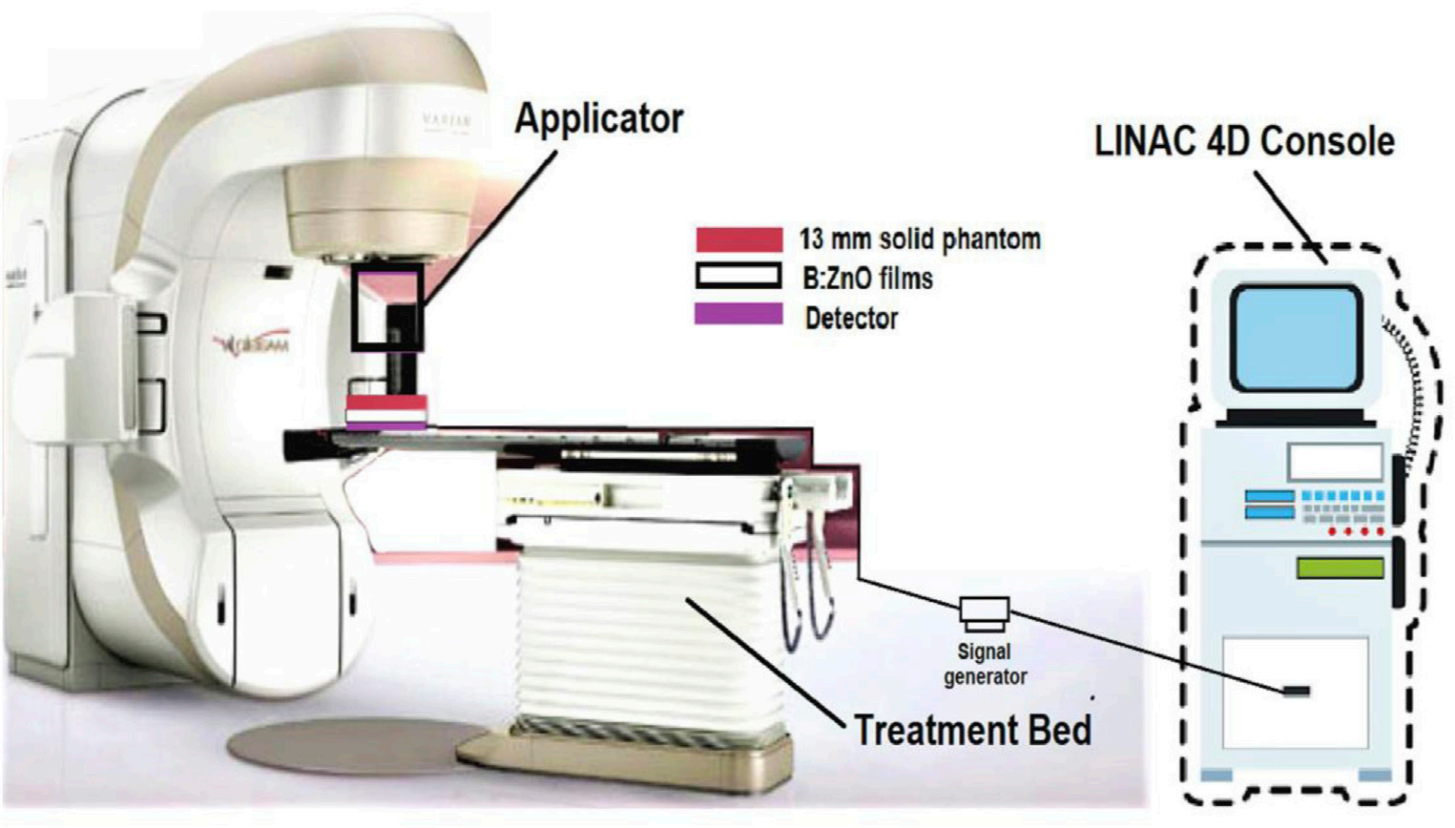
Schematic illustration of the experimental setup for radiation protection studies.

Thin films of B-doped ZnO-NSs were synthesized by using the sol-gel process and have been irradiated *via* the VARIAN^®^ (Kavun, 2019) linear accelerator. Electrons energized to 6 MeV were applied to thin films placed inside the solid phantoms. The D_max_ of 6 MeV electrons necessitated the employment of a 13 mm solid phantom for experiments. A PTW^®^ (Kavun, 2019) electron detector with a 6 × 6 cm electron field applicator was used to measure the dosage. As shown in Figure 1, the thin film samples were placed 100 cm from the gantry, and the sensor was positioned somewhere under the material samples.

## Results and Discussion

The morphology, phase study, elemental analysis of the synthesized ZnO and B-doped ZnO-NSs sample were carried out by Scanning electron microscopy (SEM), X-ray diffraction (XRD), Energy-dispersive X-ray spectroscopy (EDX), Fourier-transform infrared (FTIR), and Photoluminescence spectroscopy (PL).

**FIGURE 2:**
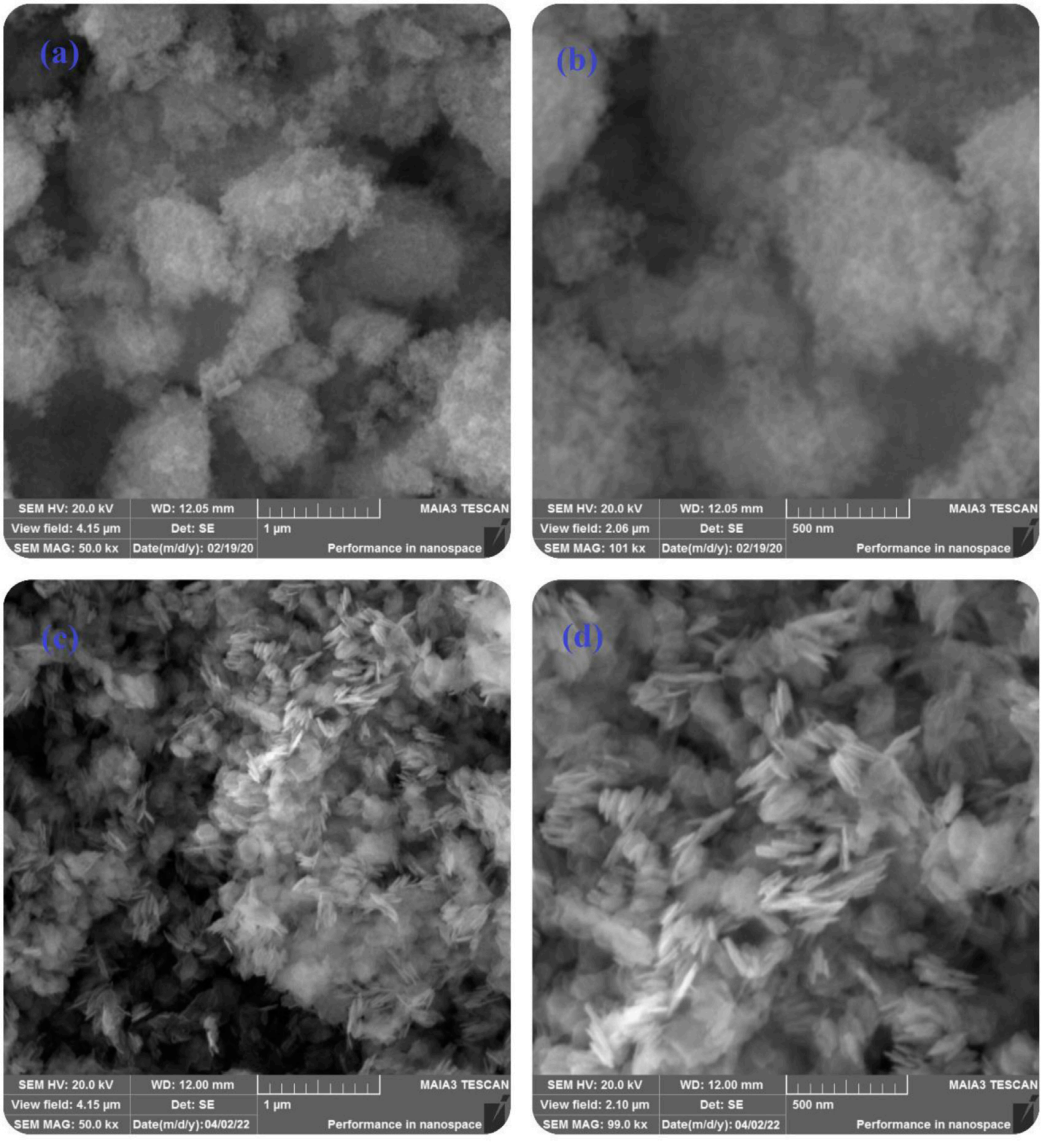
Low and high magnifications SEM micrographs of **(A,B)** pure ZnO, **(C,D)** B-doped ZnO-NSs.

The morphology of pure ZnO and B-doped ZnO-NSs synthesized by the green chemistry route was investigated by SEM. Figures 2A,B shows low, and high magnification SEM micrographs of the acquired material (pure ZnO). The micrograph in Figure 2C shows randomly aligned different shapes and morphologies of the synthesized B-doped ZnO-NSs. Smaller size nanosheets seem packed together in low magnification whereas, the larger nanosheets can be observed somehow vertically aligned. The magnified view in the high magnification SEM micrograph shown in Figure 2D clarifies the sheets-like shape and morphology of the synthesized B-doped ZnO-NSs. The vertically aligned nanosheets can be observed there among the other randomly aligned. The synthesized sample is found to have B-doped ZnO-NSs in different sizes and thicknesses. On average, the sample contained B-doped ZnO-NSs with thickness in the range of 20–80 nm.

**FIGURE 3:**
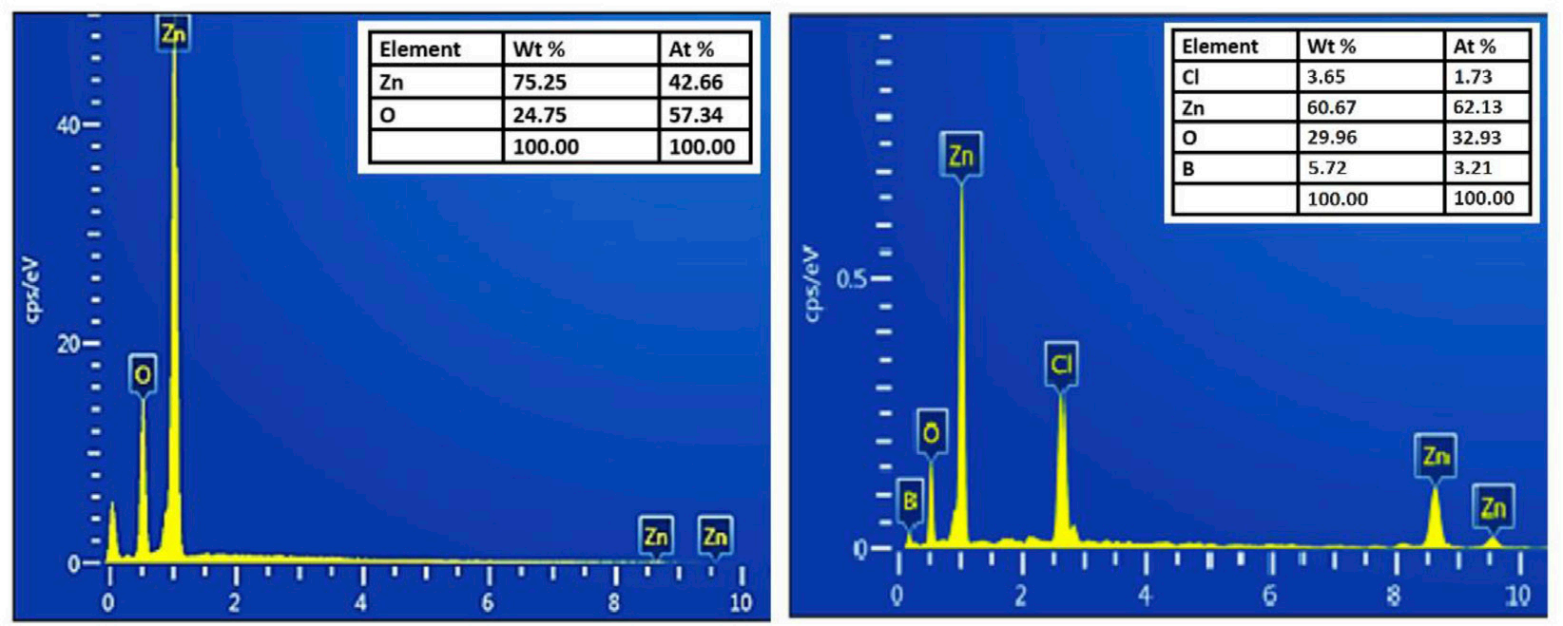
EDX spectrum of pure ZnO and B-doped ZnO-NSs.

The chemical compositional study of pure and B doped ZnO-NSs was performed by using EDX. The as-obtained EDX spectrums are shown in Figure 3. Only Zn and O were detected in the EDX spectrum of the pure sample whereas B along with Zn and O were detected in the doped ZnO-NSs samples. The presence of B ions has a considerable influence on the properties of the material. The EDX spectra also revealed that the manufactured samples included no other foreign elements except Cl.

**FIGURE 4:**
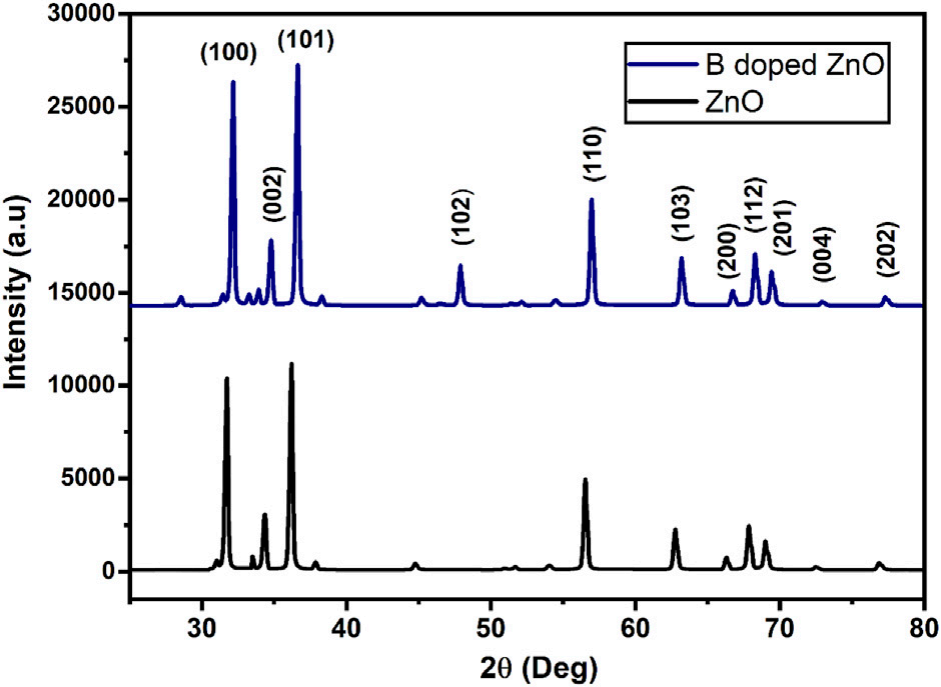
The X-ray diffraction pattern for pure (black) and B doped ZnO-NSs (blue).

The XRD (an analytical approach for determining the material’s type, its crystallite size, and phase) patterns of pure and B-doped ZnO-NSs are shown in Figure 4. The structure, phase, and crystallite size of the B-doped ZnO-NSs are driven in assistance to intensities and positions of the peaks. For the confirmation of the B-doped ZnO-NSs formation in the synthesized material, miller indices/planes were identified for every diffraction peak. The major diffraction peaks found at 31.71°, 34.39°, 36.23°, 47.70°, 62.83°, 56.59°, 66.34°, 67.89°, 68.99°, 72.61°, and 77.02° were related to the (hkl) values (miller indices/planes) of (100), (002), (101), (102), (103), (110), (200), (112), (201), (004) and (202), respectively. Crystal-like phases and the formation of a hexagonal wurtzite structure (with lattice parameters of a = 3.249 Å and c = 5.206 Å) were identified *via* X′ Pert High Score by correlating these polycrystalline ZnO-based nanostructures to the reference database (standard XRD pattern of ZnO) from ICSD file: 01-076-0704. The peaks near 33°, 38°, 45° and 54° can also be indexed to the ZnO phase, as shown in Figure 3. The low strength of these subsequent peaks shows that the obtained sample contains a very small percentage of the ZnO phase (JCPDS Card no. 21-1486). To find the crystallite size, Scherrer’s method was used.

$$D=\frac{k\lambda }{\beta \cos\theta }$$ (i)

Where D is the crystal size, λ is the wavelength of X-ray, β is the full width at half maximum of the peak in radians, and θ is the Braggs angle in radians.

The intensity of peaks was enhanced and the crystallite size of the measured (intense) peak of B-doped ZnO-NSs fell marginally from 14.6 to 13.3 nm when the doping concentration of B was increased from 0 to 15% (Kayani et al., 2020). It shows that B is successfully incorporated into the host matrix. Increased B dopant incorporation into the ZnO matrix resulted in lattice deformation, as well as lattice defects and nucleation centers (Hu and Gordon, 1992b). The nucleation site expansion and the limiting of particle growth are thus the main mechanisms underlying the generation of fine nanosheets.

**FIGURE 5:**
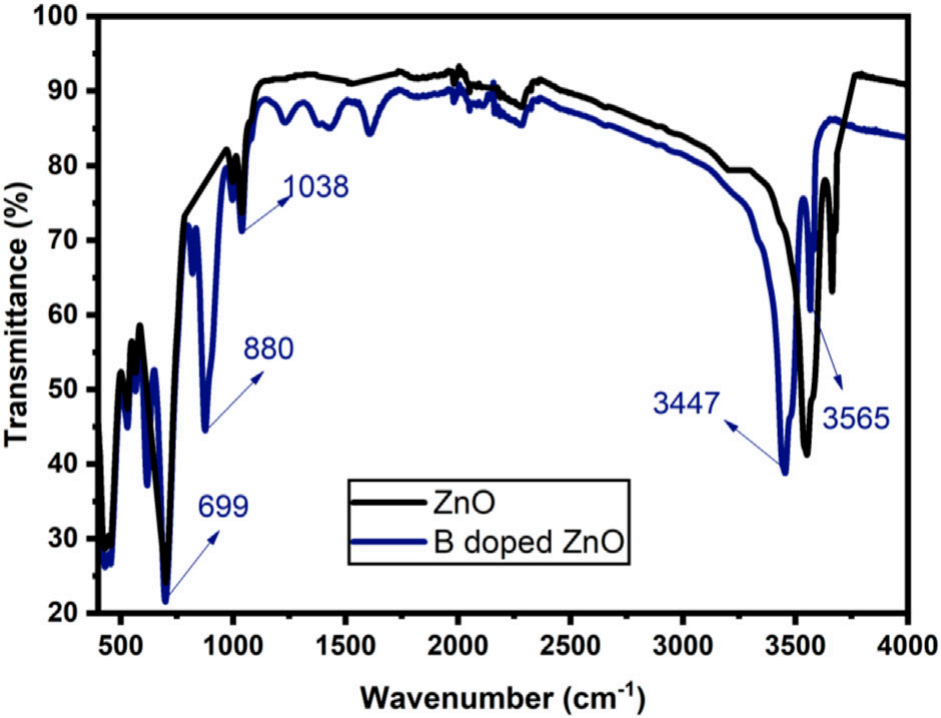
FTIR spectrum of ZnO and Boron doped ZnO-NSs.

The existence of functional groups and the incorporation of dopant ions into the host structure can be determined using FTIR spectra (Vijayaprasath et al., 2016; Khalid et al., 2021a). Transverse optical and longitudinal optical phonons (TO and LO) produced significant bands (absorption) in the area of ∼405 and ∼485 cm^−1^ as demonstrated in Figure 5. The E2h mode of ZnO was detected near 440 cm^−1^ (Jun et al., 1995). The ZnO E2h mode overlapped by a shoulder peak approximately at 559 cm^−1^, were attributed to the O—B—O boron’s deformation vibration. The existence of B—O bonds (three-coordinated) in bands visible at 495 and 418 cm^−1^ caused a clear separation of ZnO stretching modes in the host doped with boron. Meanwhile, a nicely defined band at 495 cm^−1^ shows four coordinated boron deformation vibrations (Weir, 1966). The strength of a narrow absorption band at 880 cm^−1^ linked to the growing concentration of dopant is found in ZnO-NPs (pseudo-hexagonal) doped with boron, which is attributed to the B—O bond (symmetric stretching) with boron atoms (three coordinated) (Othman et al., 2016). The absorption bands intensity at 3,447 cm^−1^ and 3,565 cm^−1^ increased for B doped ZnO-NSs. Vibrations in this region in solids are often OH stretching modes, showing how water from ambient moisture may rehydrate terminal oxygens near the sample surface. Jun et al. (Jun et al., 1995) attributed several absorption bands observed in B-doped ZnO-NS to hydrated borates produced at 20 to 100°C temperatures. The efficient integration of B into the ZnO matrix during synthesis is confirmed by enhanced performance in the measured band intensities in B doped ZnO-NSs.

**FIGURE 6:**
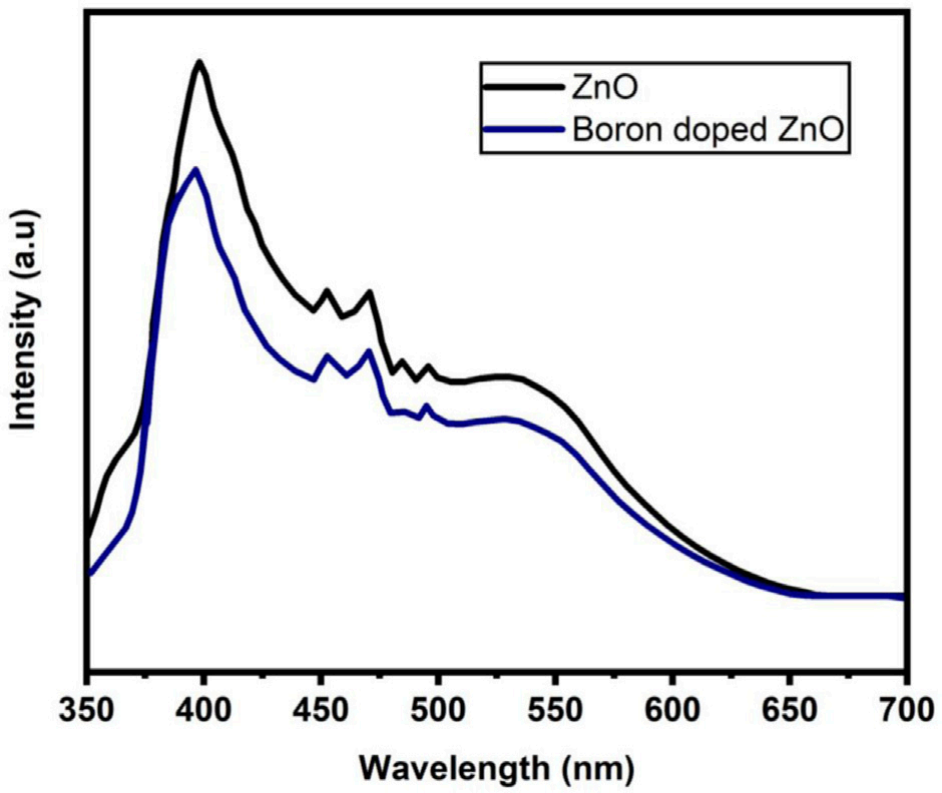
Photoluminescence (PL) spectrum of ZnO and B-doped ZnO-NSs.

The use of photoluminescence (PL) spectroscopy to explain the recombination and transport of photo-generated electron-hole pairs in semiconductors is significant. The existence of various structural defects in ZnO-NS, such as zinc and oxygen vacancies lead to varied radiative transitions among electrons from the trapping levels or conduction band and trapped or photogenerated holes can be examined by using PL measurements (Othman et al., 2016). The PL spectra of B doped ZnO-NSs at room temperature with excitation at 320 nm are shown in Figure 6. The excitonic recombination-induced UV radiation of ZnO is in phase with the near band edge emission (NBE) of ZnO, whereas the interaction of photo-generated holes with numerous structural deficiencies, such as ionized charge states of intrinsic defects, zinc vacancies, and oxygen vacancies, causes the emission of deep levels in the visible region spectrum (Das and Mondal, 2014). The PL spectra display peaks of UV emission at 380 and 395 nm. Accordingly, the spectrum has a strong blue band at 469 nm, a violet-blue band at 449 nm, a moderate blue-green emission at 495 nm, a greenish-blue emission at 482 nm, and a broad peak from 500-650 nm. A peak at 380 nm in UV light indicates normal NBE or exciton emission from free exciton recombination (Xia et al., 2011). Surface states (VZn) or band tail states in ZnO are responsible for the peak at 395 nm (Palni et al., 2007). The optical centers related to impurities including intrinsic defects are commonly detected in the wavelength range of 450–650 nm in the DLE area (Thapa et al., 2016). The faint blue emissions are primarily caused by surface imperfections in ZnO NSs (Wang and Gao, 2004). The electron transformations from the interstitial Zn to the intrinsic defect VZn occurred in the bright blue band at 469 nm. Furthermore, at 543 nm, a green emission known as the DLE can be seen, which is often ascribed to single ionized oxygen vacancies (V0+) (Shi et al., 2011). Figure 6 clearly shows that the intensity of PL spectra has reduced as a result of the addition of B to ZnO. Based on the observation, The B^3+^ ion has been transferred to the lattice sites effectively into the host matrix (ZnO). The B-doped ZnO has the smallest surface defect, as seen by the weak green emission on the PL graph (Wang et al., 2017).

**FIGURE 7:**
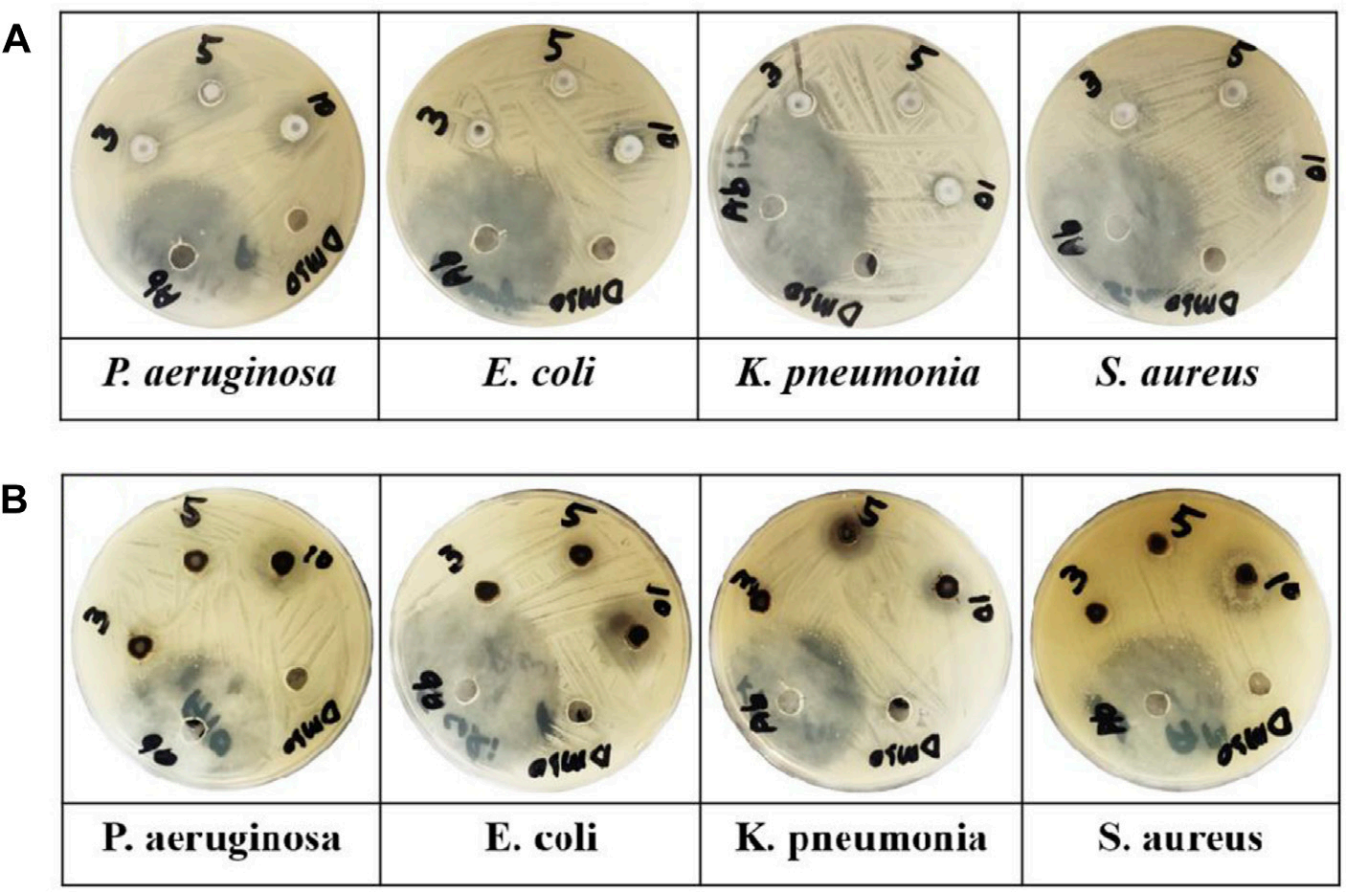
**(A)** ZnO and **(B)** B-doped ZnO-NSs applied against various microbe (*P. aeruginosa, K. pneumonia, E. coli*, and *S. aureus*) in Petri plates.

**FIGURE 8:**
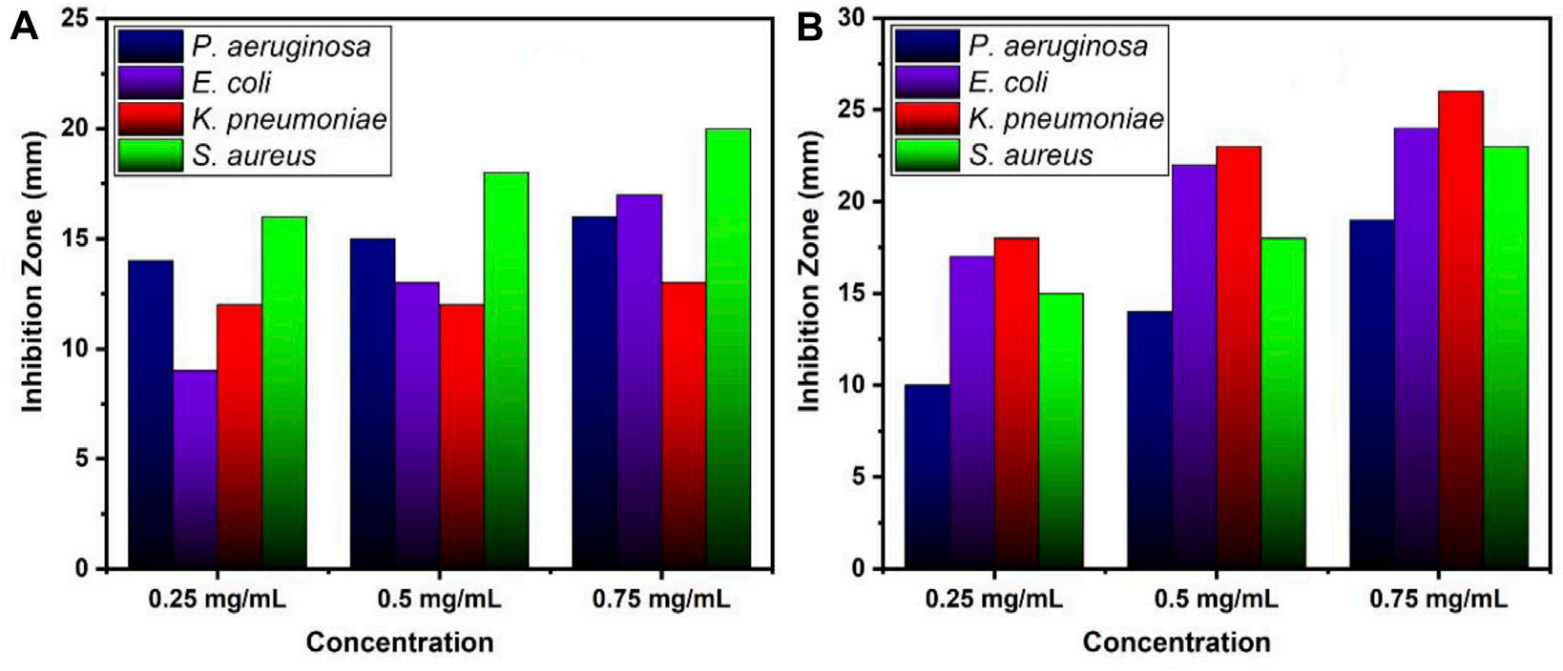
Zone of inhibition values (mm) for various concentrations of **(A)** ZnO and **(B)** B doped ZnO-NSs.

Table 1 and Figure 7 illustrate the antibacterial efficiency of ZnO and B-doped ZnO against various bacterial isolates. The findings revealed that doping B enhanced antibacterial activity. The increase in antibacterial activity could be due to one of two factors. B-doped ZnO small particle size makes it possible for them to enter bacterial cell walls more easily. The antibacterial activity will be damaged and reduced due to the agglomeration of nano crystallites. In this study, a smaller particle size resulted in the release of high concentrations of B ions into the environment. The results show that due to smaller particle size, doping of B raised the antibacterial action. Antibacterial activity of pure ZnO shows 16 and 17 mm zone of inhibition for *P. aeruginosa* (ATCC^®^ 10145) and *E. coli* (ATCC^®^ 33876) while for *K. pneumoniae* (ATCC^®^ BAA-1144) and *S. aureus* (ATCC^®^ 11632) ZnO shows 13 and 20 mm zone of inhibition. B doped ZnO-NSs was shown to be exceptional against both *E. coli* (ATCC^®^ 33876) and *K. pneumonia* (ATCC^®^ BAA-1144) tested bacterial strains. *E. coli* (ATCC^®^ 33876) had a 24 mm zone of inhibition, while *K. pneumonia* (ATCC^®^ BAA-1144) had a 26 mm zone of inhibition as demonstrated in Figures 8A,B.

**TABLE 1:** Information of bacterial isolates and other experimental parameters.

Bacteria			ZnO			B Doped ZnO		
			0.25 mg/ml	0.5 mg/ml	0.75 mg/ml	0.25 mg/ml	0.5 mg/ml	0.75 mg/ml
Gram negative	*P. aeruginosa*	Inhibition zone (mm)	14 ± 0.12	15 ± 0.11	16 ± 0.18	10 ± 0.12	14 ± 0.13	19 ± 0.15
	*E. coli*		09 ± 0.17	13 ± 0.14	17 ± 0.23	17 ± 0.15	22 ± 0.10	24 ± 0.22
	*K. pneumoniae*		12 ± 0.11	12 ± 0.12	13 ± 0.21	18 ± 0.14	23 ± 0.19	26 ± 0.21
**Gram positive**	*S. aureus*		16 ± 0.15	18 ± 0.20	20 ± 0.19	15 ± 0.14	18 ± 0.17	23 ± 0.16

**FIGURE 9:**
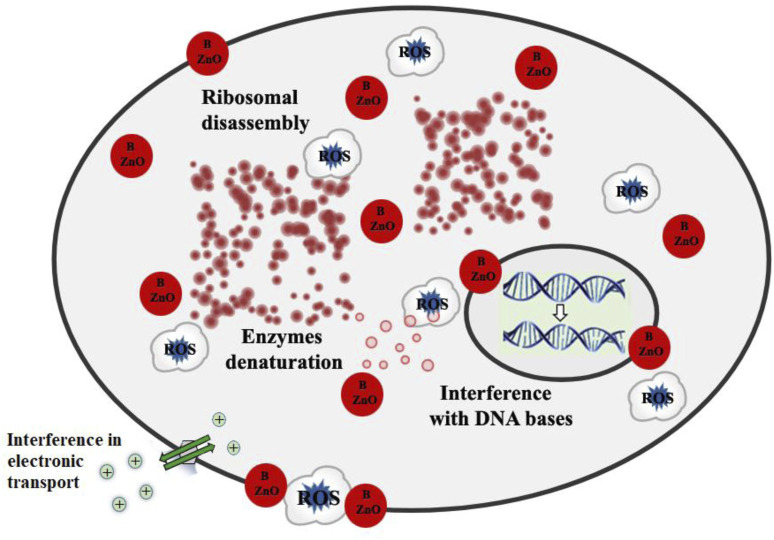
Schematic illustration for detailed antibacterial mechanism of pure and B doped ZnO-NSs.

This study found a broad range of antibacterial efficacy against various types of bacteria. These findings revealed that the antibacterial activity of ZnO was influenced by dopant types. The complete mechanism of the influence of synthesized B doped ZnO NSs on various microbial strains is demonstrated in Figure 9. In our previous works, we have studied the antibacterial efficiency of pure ZnO, Cu-doped ZnO (Khalid et al., 2021b), and Co-doped ZnO (Khalid et al., 2021c) nanostructures. The comparison of the higher concentration of those results and the current results are given in Table 2, from which it can easily be observed that the anti-bactericidal efficiency of B-doped ZnO-NSs is higher than pure, Cu-doped, and Co-doped ZnO nanostructured material (Khalid et al., 2021b; Khalid et al., 2021c).

**TABLE 2:** Antibacterial efficiency of B-doped ZnO-NSs in comparison with pure, Cu-doped, and Co-doped ZnO.

Bacteria		ZnO	Cu Doped ZnO (Khalid et al., 2021b)	Co Doped ZnO (Khalid et al., 2021c)	B Doped ZnO
*E. Coli*	Inhibition zone (mm)	1 mg/ml	1 mg/ml	1 mg/ml	0.75 mg/ml
		14 ± 0.28	18 ± 0.36	17 ± 0.34	24 ± 0.22
*K. pneumoniae*		15 ± 0.3	17 ± 0.34	19 ± 0.38	26 ± 0.21
*S. aureus*		13 ± 0.26	24 ± 0.48	15 ± 0.3	23 ± 0.16
*S. pyogenes*		9 ± 0.18	20 ± 0.4	16 ± 0.32	---
*P. aeruginosa*		---	---	---	19 ± 0.15

**FIGURE 10:**
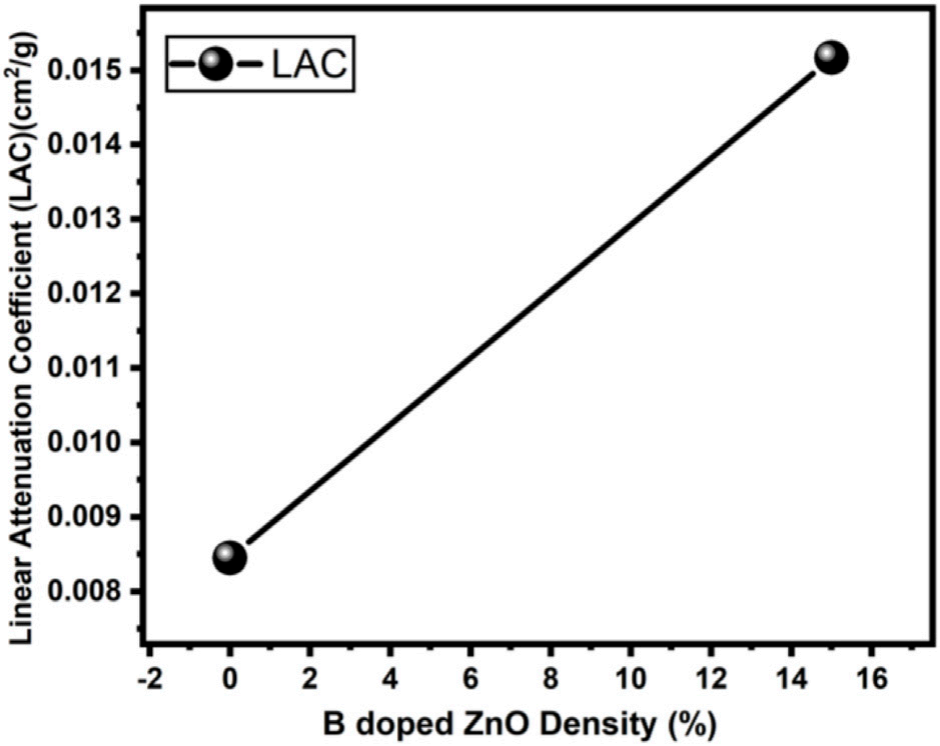
Linear Attenuation Coefficient of B doped ZnO (0 and 15%).

**FIGURE 11:**
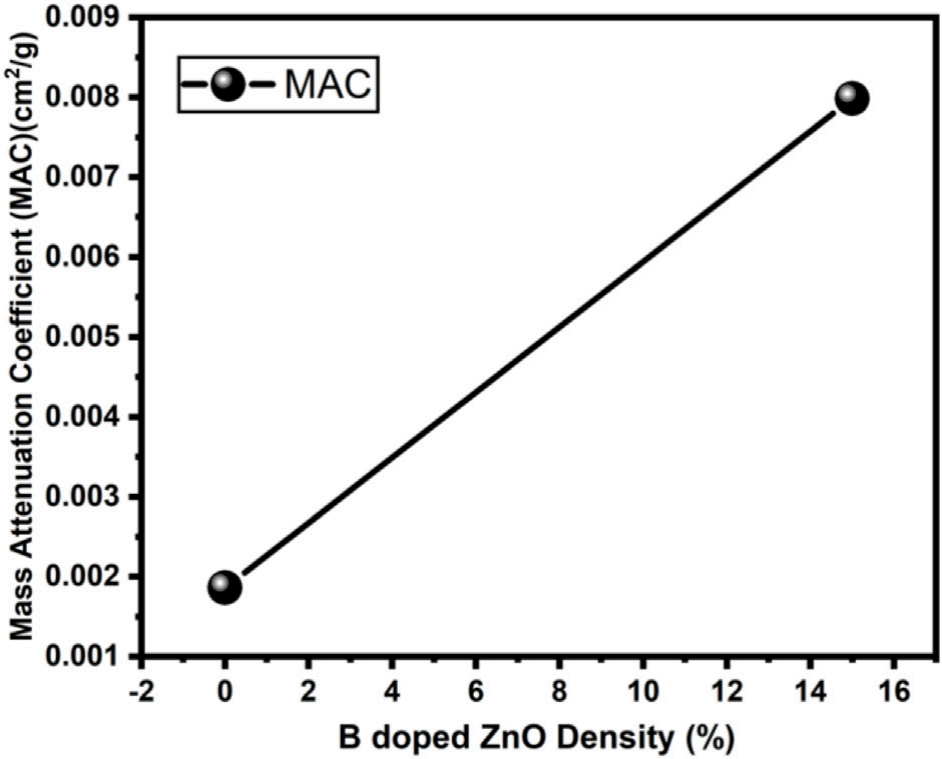
Mass attenuation Coefficient of B doped ZnO (0 and 15%).

**FIGURE 12:**
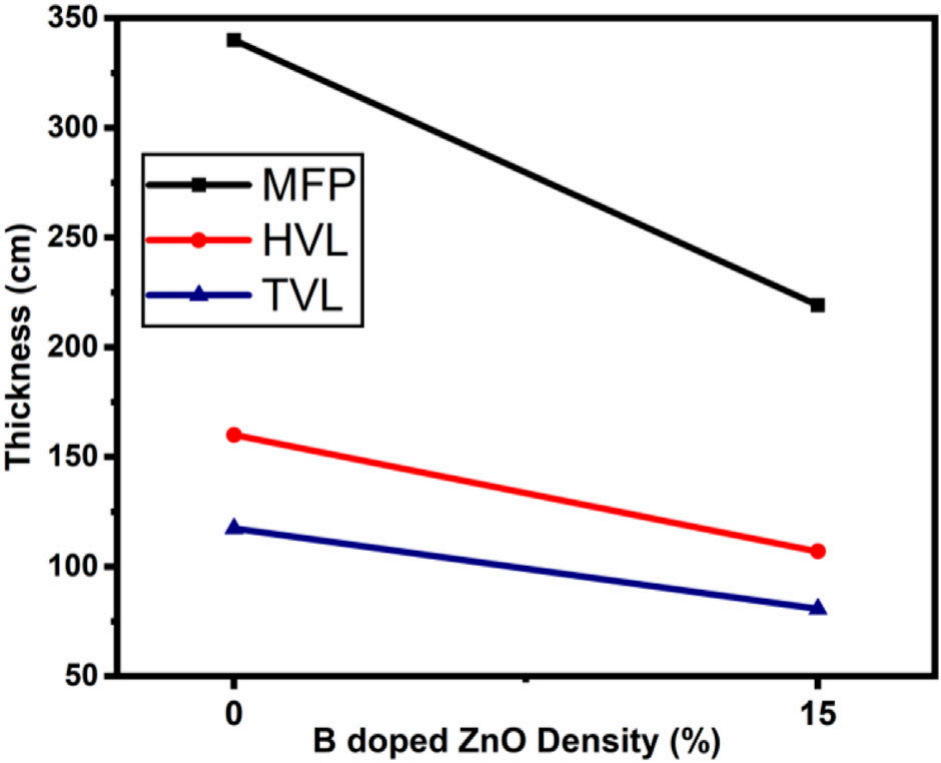
Mean Free Path, Half-value Layer and One-tenth Value Layer of B doped ZnO (0 and 15%).

The radiation protection/shielding efficiency of ZnO and B doped ZnO thin films can be determined by using linear attenuation measurements. Boron has been doped into these coating materials. 6 MeV electrons were used to irradiate the sample. Table 2 shows the results of linear attenuation (μ), mass attenuation (µm) coefficients, one-tenth value layer, half-value layer, and mean free path of pure and doped ZnO thin films. As can be shown in Table 3B doping has significantly contributed to these values. The highest value of linear attenuation coefficient (0.0151 ± 0.0011 cm^−1^) was achieved for boron-doped ZnO thin film. Figure 10 shows the linear attenuation coefficient values that have been fitted linearly. Figure 11 depicts the mass attenuation coefficient of B doped ZnO thin films. For ZnO-coated thin films, the lowest value is 0.00186 ± 0.00035 cm^2^/g. The value grows with the B incorporation into the host matrix. At B-doped ZnO thin film, the mass attenuation coefficient is maximum at 0.00799 ± 0.00047 cm^2^/g. The One-Tenth Values (TVL), Mean Free Path (MFP), and Half-Value Layer (HVL), of pure and B-doped ZnO thin films, are presented in Figure 12. Depending on the proportion of dopant (boron) in ZnO thin films, the Half value layer has altered from 160.03 cm to 106.99 cm^−1^. The one-tenth layer value has also changed, going from 117.47 cm to 80.8 cm^−1^.

**TABLE 3:** Various parameters (LAC, MAC, MFP, HVL, TVL) to analyze the radiation shielding efficiency of B doped ZnO.

Sample	Composition %		LAC (cm^−1^)	MAC (cm^2^/g)	MFP (cm)	HVL (cm)	TVL (cm)
	ZnO	Boron					
1	100	0	0.00845	0.00186	340.09	160.03	117.47
2	85	15	0.01517	0.00799	219.12	106.99	80.8

Finally, the mean free path of B-doped ZnO thin films has decreased from 340.09 to 219.12 cm. These values decrease as the amount of boron content in the thin film increases. In addition, future studies in the same field can help in building an alternative experimental setup, where the material’s absorption, their effects, and the interactions between the various particles can be studied (Maaza et al., 2006; Mtshali et al., 2013). Thus, the radiation shielding properties of nanomaterials will be best known with the additional studies to be done (Izerrouken et al., 2011; Ahmad et al., 2022; Khalid et al., 2022).

## Conclusion

*Phyllanthus emblica* (gooseberry) leaves extract in the green chemistry method can effectively be utilized in the synthesis of B-doped ZnO-NSs. A comparative study of B-doped ZnO-NSs showed a stimulating effect of B-doping on radiation protection and anti-bacterial characteristics. All of the microorganisms tested were found to be inhibited by B-doped ZnO-NSs. The inhibitory impact is dose-dependently increased. Gram-negative isolates are more sensitive to B-doped ZnO-NSs than gram-positive isolates. The antibacterial activity of ZnO-NSs is greatly enhanced by the addition of B-dopant. According to this study, doping appears to be a successful technique for the synthesis of the most effective antibacterial agent. As per the radiation shielding characteristics of the material, the linear attenuation coefficient is enhanced due to an increase in the amount of boron (0 to 15%) in the material. The one-tenth value layer, half-value layer, mass attenuation coefficient, and mean free path of B-doped ZnO-NSs all behaved in the same manner. These findings suggest that B-doped ZnO-NSs can be employed in radiation shielding applications in the modern world.

## Data Availability

The original contributions presented in the study are included in the article/Supplementary Material, further inquiries can be directed to the corresponding authors.
